# Comprehensive evaluation of compound Kushen injection combined with zoledronic acid in treating bone metastasis cancer pain based on meta-analysis and decision tree model

**DOI:** 10.3389/fpain.2024.1512925

**Published:** 2025-01-17

**Authors:** Xi Zhao, Tianwei Meng, Kaiqiang Wang, Xi Yan, Yuqiang Liu, Xinghua Li

**Affiliations:** ^1^ Department of Pharmacy, Changzhi People’s Hospital Affiliated to Changzhi Medical College, Changzhi, China; ^2^ Graduate School, Heilongjiang University of Chinese Medicine, Harbin, China; ^3^ School of Pharmacy, Changzhi Medical College, Changzhi, China

**Keywords:** meta-analysis, decision tree, compound Kushen injection, zoledronic acid, bone metastasis

## Abstract

**Objective:**

To evaluate the safety, efficacy, and cost-effectiveness of combining Compound Kushen Injection (CKI) with zoledronic acid in the treatment of bone metastasis-induced cancer pain in malignant tumors.

**Methods:**

A comprehensive search of Chinese and English databases identified randomized controlled trials (RCTs) investigating CKI combined with zoledronic acid for bone metastases in malignancies. Methodological quality assessments were performed on all included studies, and a meta-analysis was conducted using RevMan 5.4.1 software. A cost-effectiveness analysis from the perspective of China's healthcare system employed a decision tree model to evaluate the short-term economic impact of the two treatment regimens. Sensitivity analyses assessed the robustness of the results.

**Results:**

Fourteen studies involving 1,269 patients were included in the meta-analysis. The results demonstrated that CKI combined with zoledronic acid was more effective than zoledronic acid alone in treating bone metastatic cancer pain (OR = 3.43, 95% CI: 2.51–4.67, *P* < 0.0001), with no significant difference in adverse reactions between the two groups. Incremental cost-effectiveness ratio (ICER) analysis revealed that the combination therapy incurred an additional cost of ¥18,863.16 for each unit of effect gained compared to zoledronic acid alone. Sensitivity analyses indicated stable results, showing that under the assumption of a willingness-to-pay threshold set at the average per capita disposable income in 2023, the combination of CKI and zoledronic acid was more cost-effective than zoledronic acid alone in treating bone metastatic cancer pain.

**Conclusion:**

Compared with zoledronic acid alone, the combination of CKI and zoledronic acid offers superior efficacy, high safety, and better cost-effectiveness in the treatment of bone metastasis-induced cancer pain in malignant tumors.

## 1 Introduction

Bone metastasis is a common and severe complication of malignant tumors, occurring when cancer cells spread from their primary site to bone via hematogenous routes (1). It is most frequently observed in patients with advanced breast, lung, prostate, and renal cancers (2). The skeletal system provides a conducive environment for metastatic tumor cells, leading to a range of skeletal-related events. Bone metastases can cause significant morbidity, including severe bone pain, pathological fractures, hypercalcemia, anemia, and spinal cord compression, all of which profoundly diminish patients' quality of life and increase mortality rates (3). Among these complications, cancer-induced bone pain is one of the most challenging symptoms to manage, significantly affecting patient functionality, emotional well-being, and overall quality of life. Effective management of bone pain is thus a critical component of palliative care in oncology.

Current therapeutic approaches for bone metastasis primarily focus on symptom palliation, inhibition of tumor progression within bone, and prevention of skeletal-related events. Bisphosphonates, such as zoledronic acid, are potent inhibitors of osteoclast-mediated bone resorption and are commonly used to reduce bone pain and prevent fractures (4). However, their efficacy in pain control is sometimes limited, and patients may experience adverse effects such as osteonecrosis of the jaw and renal toxicity (5). Additionally, the high cost of long-term bisphosphonate therapy poses economic challenges for patients and healthcare systems.

In recent years, Traditional Chinese Medicine (TCM) has gained attention as an adjunctive treatment in cancer care due to its holistic approach and lower side-effect profile (6). Compound Kushen Injection (CKI), derived from the medicinal herbs Kushen (*Radix Sophorae Flavescentis*) and Baituling (*Rhizoma Smilacis Glabrae*), has been used in TCM to “clear heat”, “detoxify”, alleviate pain, and disperse masses. Pharmacological studies have demonstrated that CKI exhibits anti-tumor, anti-angiogenic, anti-inflammatory, and immunomodulatory effects (7). It can inhibit cancer cell proliferation, induce apoptosis, and modulate pain pathways by affecting neurotransmitter release and receptor expression (8). Combining CKI with conventional therapies like zoledronic acid may offer synergistic effects, enhancing analgesia, inhibiting tumor growth, and improving patients' immune function.

Despite the promising potential of CKI, rigorous scientific evaluation is needed to substantiate its clinical benefits and economic value when combined with standard treatments. Therefore, this study aims to conduct a comprehensive meta-analysis of randomized controlled trials (RCTs) to assess the efficacy and safety of CKI in combination with zoledronic acid for the treatment of bone metastasis-induced cancer pain. Additionally, recognizing the importance of cost-effectiveness in healthcare decision-making, we utilize a decision tree model to evaluate the short-term economic impact of the combined therapy from the perspective of China's healthcare system. Sensitivity analyses are performed to ensure the robustness of the results. By integrating evidence-based medicine principles with economic evaluation tools, this study seeks to provide robust evidence to guide clinical practice and inform health policy decisions regarding the management of bone metastatic cancer pain. Ultimately, we aim to determine whether the addition of CKI to standard therapy offers a superior therapeutic strategy that is both clinically effective and economically viable.

## 2 Materials and methods

### 2.1 Meta-analysis

A systematic review and meta-analysis were conducted to evaluate the efficacy, safety, and cost-effectiveness of CKI combined with zoledronic acid in the treatment of bone metastatic cancer pain in patients with malignant tumors. The study adhered to the Preferred Reporting Items for Systematic Reviews and Meta-Analyses (PRISMA) guidelines.

#### 2.1.1 Literature search strategy

A comprehensive literature search was performed across multiple electronic databases, including PubMed, EMBASE, Cochrane Library, CNKI, VIP Database, and Wanfang Data, covering all publications up to April 10, 2024. The search strategy employed a combination of Medical Subject Headings (MeSH) terms and free-text keywords related to the interventions and the condition of interest. For English-language databases, terms such as “Compound Kushen Injection”, “Zoledronic Acid”, “Bone Metastasis”, “Bone Cancer Pain”, “Osseous Metastasis”, “Ostealgia”, and “Malignant Tumor” were used. Equivalent terms were utilized for Chinese-language databases. No language restrictions were applied to maximize the retrieval of relevant studies. Additionally, reference lists of all identified articles were manually searched to identify any additional eligible studies.

#### 2.1.2 Inclusion and exclusion criteria

Studies were included if they met the following criteria: they were randomized controlled trials involving patients with pathologically confirmed malignant tumors and bone metastasis diagnosed through imaging modalities such as electron beam computed tomography (ECT) bone scan, computed tomography (CT), or magnetic resonance imaging (MRI), and exhibiting clinical symptoms of bone pain. The experimental group received CKI in combination with zoledronic acid, while the control group received zoledronic acid alone. The primary outcome measured was the overall pain relief rate, and secondary outcomes included the incidence of adverse events such as fever, rash, and gastrointestinal reactions. Studies were excluded if they were non-randomized controlled trials, observational studies, case reports, reviews, or editorials, or if they lacked primary outcome data or if valid information could not be extracted.

#### 2.1.3 Literature screening and data extraction

Study selection and data extraction were performed independently by two reviewers to minimize bias and errors. Initially, the titles and abstracts of all retrieved articles were screened to identify potentially eligible studies. Full-text articles were then obtained for studies that appeared to meet the inclusion criteria or when eligibility was unclear from the abstract. The full texts were assessed independently to determine final inclusion. Discrepancies between reviewers were resolved through discussion or, if necessary, consultation with a third reviewer to reach a consensus.

#### 2.1.4 Quality assessment and risk of bias evaluation

The methodological quality and risk of bias of the included studies were assessed independently by the two reviewers using the Cochrane Collaboration's Risk of Bias Tool (version 5.1.0). The assessment covered various domains, including selection bias (random sequence generation and allocation concealment), performance bias (blinding of participants and personnel), detection bias (blinding of outcome assessment), attrition bias (incomplete outcome data), reporting bias (selective reporting), and other potential sources of bias. Each domain was judged as having a low, high, or unclear risk of bias based on the information provided in the studies. Any disagreements in the assessment were resolved through discussion or by involving a third reviewer to ensure an objective evaluation.

#### 2.1.5 Statistical analysis and meta-analysis

Statistical analyses were performed using Review Manager software version 5.4. For dichotomous outcomes, odds ratios (ORs) with 95% confidence intervals (CIs) were calculated to measure the effect size. Heterogeneity among studies was assessed using the Chi-squared (χ^2^) test and quantified with the *I*^2^ statistic. An *I*^2^ value of 50% or less, combined with a *P*-value greater than 0.10, was considered to indicate low statistical heterogeneity, and a fixed-effect model was employed for the meta-analysis. If significant heterogeneity was detected (*I*^2^ greater than 50% or *P*-value of 0.10 or less), a random-effects model was used to pool the data. Potential publication bias was evaluated through visual inspection of funnel plots when a sufficient number of studies (generally more than ten) were included in the analysis.

### 2.2 Pharmacoeconomic evaluation

#### 2.2.1 Target population and interventions

For the pharmacoeconomic evaluation, the target population consisted of individuals with bone metastases from malignant tumors who presented with bone pain symptoms, aligning with the patients included in the meta-analysis. The interventions compared in the cost-effectiveness analysis were as follows: the control group received zoledronic acid injection at a dose of 4 mg, administered intravenously once every 14 days for a total of two doses; the experimental group received the same regimen of zoledronic acid combined with CKI at a dose of 20 ml per day, administered intravenously for 28 consecutive days.

#### 2.2.2 Model structure

**Figure 1:**
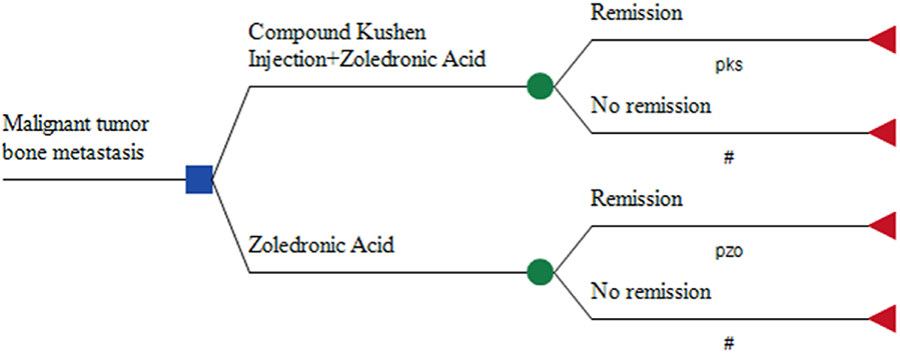
Decision tree model structure diagram.

Given the short duration of the clinical studies included and the lack of long-term survival data, a decision tree model was employed for the cost-effectiveness evaluation. This model utilized the results of the meta-analysis to compare the short-term economic benefits of the two treatment regimens (9). The outcome measure was treatment effectiveness, coded as 1 for effectiveness and 0 for ineffectiveness. The study duration was 28 days. The model was constructed using TreeAge Pro 2019 software, with the specific structure illustrated in Figure 1.

#### 2.2.3 Model parameters

The total effective rate of pain relief was used as the primary measure of treatment efficacy. Based on the forest plot results from the meta-analysis, a weighted calculation of the total effective rate for both groups was performed. Cost-effectiveness evaluation was conducted from the healthcare system's perspective, considering only direct medical costs. According to the meta-analysis results, if there was no statistically significant difference in the incidence of adverse reactions, the costs of managing adverse events were not included.

Drug prices were obtained from Yaozhi.com (https://www.yaozh.com/) and adjusted by ±20% for sensitivity analysis. CKI was administered in 5 ml vials, with a daily dosage of 4 vials; the usage range was varied between 2 and 6 vials for sensitivity analysis. The number of treatment days for zoledronic acid and CKI was also subjected to ±20% variation for sensitivity analysis.

#### 2.2.4 Evaluation method

Using the constructed decision tree model, the ICER was calculated through cost-effectiveness analysis and compared with the willingness-to-pay (WTP) threshold. If the ICER was lower than the WTP threshold, the combination of CKI and zoledronic acid was considered cost-effective; otherwise, it was not. Based on the 2023 national average disposable income (¥39,218) (10), ¥39,218 was set as the WTP threshold in this study.

#### 2.2.5 Sensitivity analysis

Both one-way sensitivity analysis and probabilistic sensitivity analysis were conducted to test the robustness of the model. One-way sensitivity analysis calculated the effect of each parameter's variation on the ICER within the upper and lower limits of the pre-specified range. If the 95% CI for a parameter was known, the CI was used as the range; if not, a ±20% variation around the mean was used. The results were illustrated using a tornado diagram.

Probabilistic sensitivity analysis assessed the combined impact of all parameter variations on the cost-effectiveness of both treatment options. Based on the distribution characteristics of the parameters [Gamma distribution for cost data, Beta distribution for utility values and event rates, and Uniform distribution for drug administration days (11)], Monte Carlo simulation was performed 1,000 times. The results were displayed as cost-effectiveness scatter plots and cost-effectiveness acceptability curves.

## 3 Results

### 3.1 Meta-analysis results

#### 3.1.1 Literature screening results and basic characteristics of included studies

**Figure 2:**
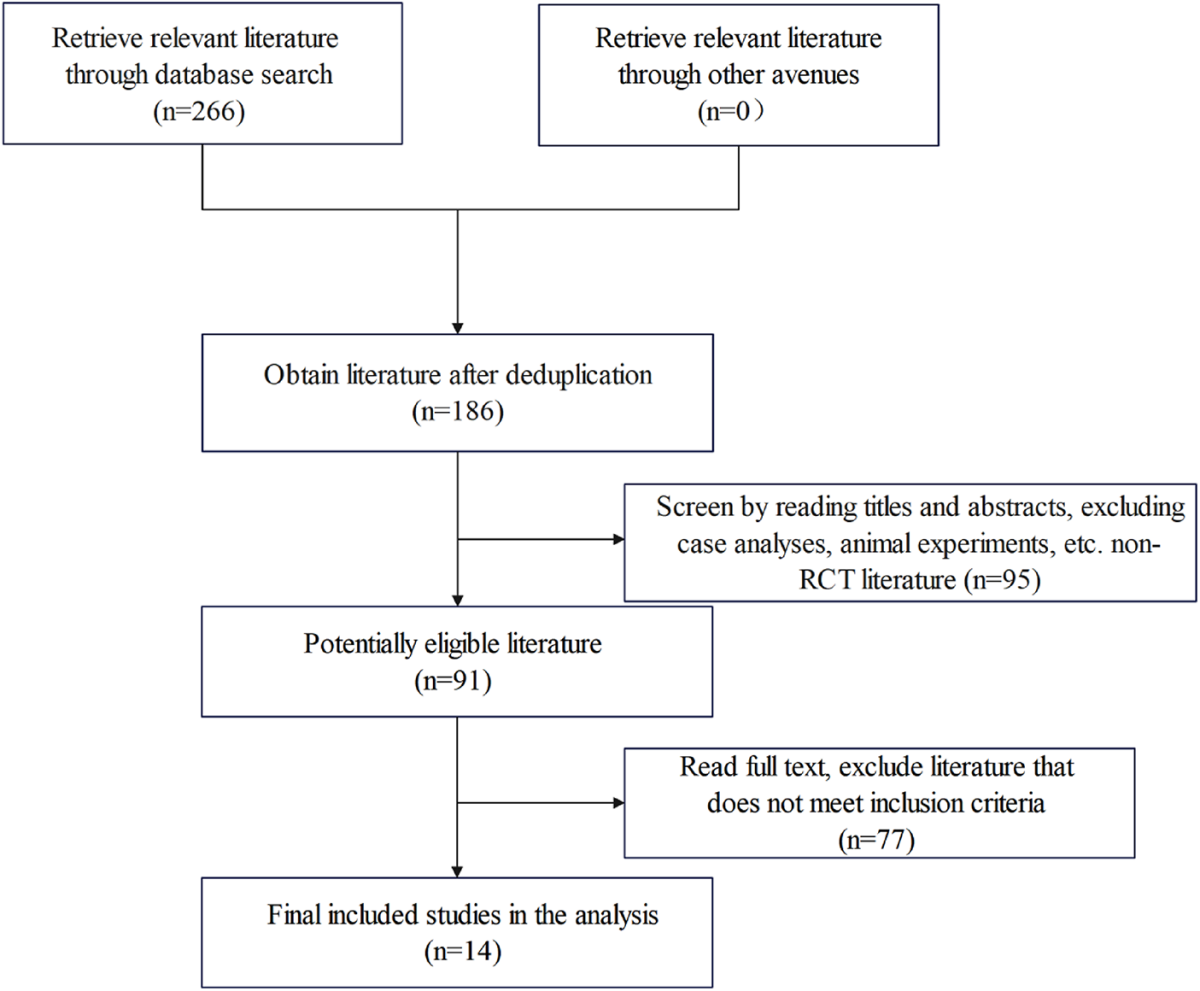
Literature retrieval flowchart.

After a comprehensive database search and full-text screening, 14 RCTs were included in the analysis the specific screening steps shown in Figure 2. These 14 studies involved a total of 1,269 patients, with 656 in the experimental group and 613 in the control group. A total of 704 males and 565 females were included, with ages ranging from 30 to 84 years. The main types of cancers involved included breast cancer, lung cancer, stomach cancer, nasopharyngeal carcinoma, among others. In all included studies, there were no statistically significant differences in age, gender, tumor type, and other basic conditions between the experimental and control groups, ensuring baseline comparability. Thirteen studies (12–24) reported the overall pain relief rate, seven studies (12–17, 25) reported the incidence of fever, eight studies (12, 13, 15–19, 25) reported gastrointestinal adverse reactions such as nausea and vomiting, and five studies (13, 16–18, 21) reported skin adverse reactions such as rash and itching.

**Figure 3:**
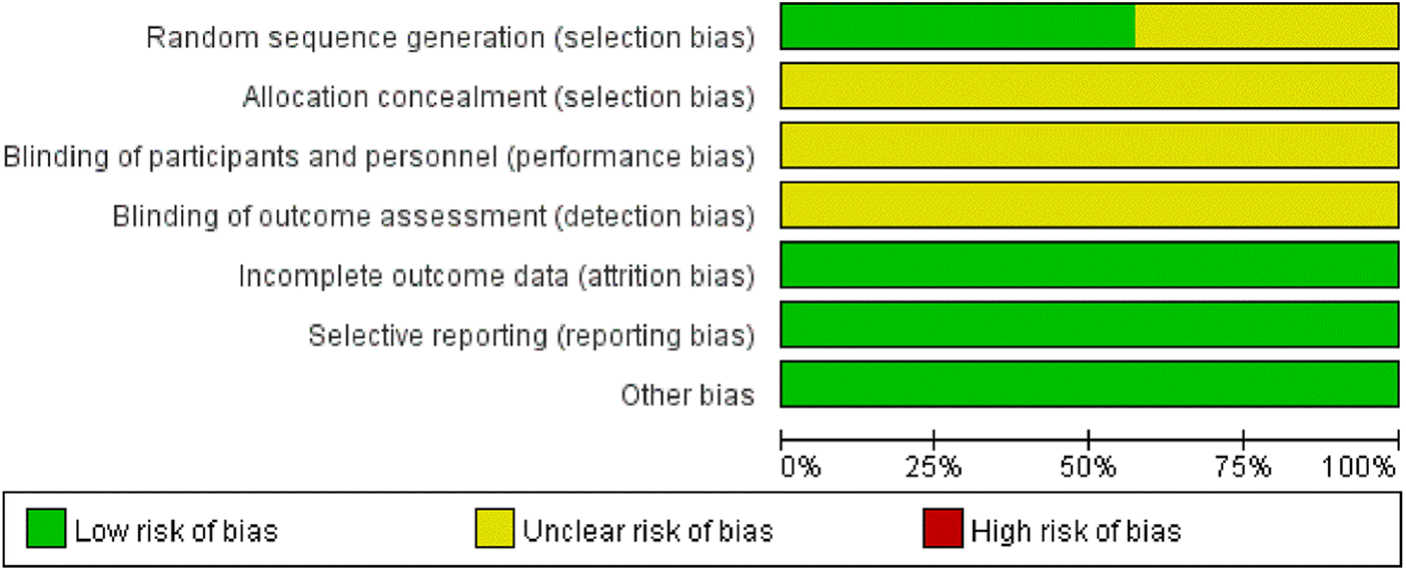
Risk of bias graph.

**Figure 4:**
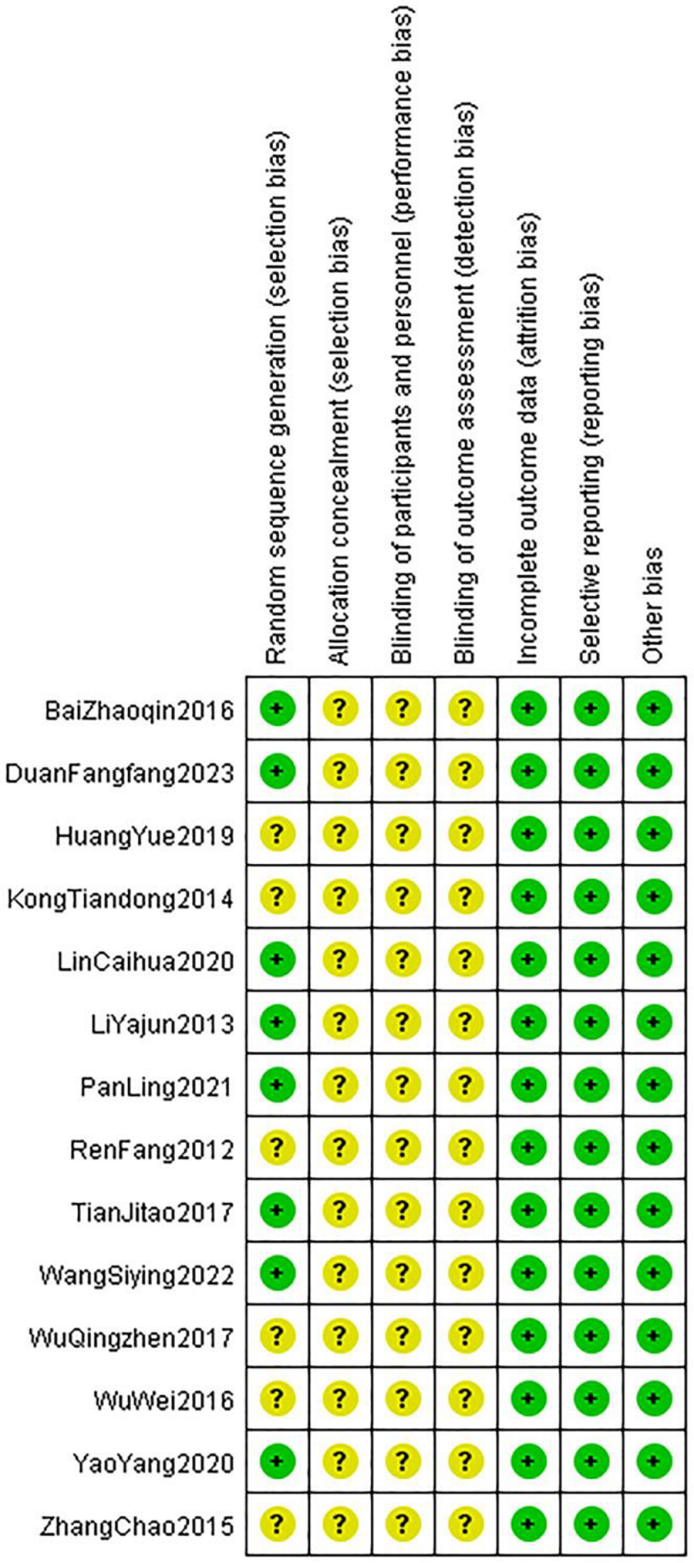
Risk of bias summary.

Regarding randomization methods, seven studies reported using a random number table, one used a lottery method, and the rest did not specify the randomization process. None of the included studies mentioned whether blinding was implemented or whether allocation concealment was described, leading to an overall low-quality rating for these studies. The basic information of the included studies is presented in Table 1, and the risk of bias assessment results are shown in Figures 3, 4.

**Table 1:** Basic information of the included studies.

Author	Type of malignancy	Case (T/C)	Sex (Female/Male)	Average age (T/C)	Intervention study		Course of treatment (day)	Outcome indicators	Stochastic method
					Test group	Control group			
Duan Fangfang 2023	Breast cancer	40/40	80/0	61.65 ± 7.63/62.10 ± 5.78	zoledronic acid+CKI	zoledronic acid	28*4	①②	Random number table
Wang Siying 2022	Lung cancer	41/41	33/49	57.61 ± 6.31/58.04 ± 6.26	zoledronic acid+CKI	zoledronic acid	28*4	①②	Random number table
Pan Ling 2021	Lung cancer	50/50	37/63	54.06 ± 7.11/53.82 ± 7.56	zoledronic acid+CKI	zoledronic acid	28	①	Random number table
Lin Caihua 2020	Lung cancer	45/45	35/55	56.28 ± 3.16/56.45 ± 3.08	zoledronic acid+CKI	zoledronic acid	30	②	Random number table
Yao Yang 2020	Lung cancer, breast cancer, colon cancer, etc.	35/35	31/39	54 ± 6.1/55.2 ± 7.5	zoledronic acid +CKI	zoledronic acid	28*4	①	Random number table
Huang Yue 2019	Lung cancer	55/58	43/70	61.56 ± 8.57/60.93 ± 8.77	zoledronic acid+CKI	zoledronic acid	28	①②	Not described
Tian Jitao 2017	Lung cancer, breast cancer, stomach cancer, etc.	50/50	35/65	55.31 ± 12.38	zoledronic acid+CKI	zoledronic acid	28	①②	Random number table
Kong Tiandong 2014	Lung cancer, breast cancer, etc.	107/69	59/117	no significant difference	zoledronic acid+CKI	zoledronic acid	28	①②	Not described
Ren Fang 2012	Lung cancer, breast cancer, etc.	35/29	26/38	63.81 ± 6. 93/64.37 ± 7. 02	zoledronic acid+CKI	zoledronic acid	28	①②	Not described
Wu Wei 2016	Breast cancer, lung cancer, prostate cancer, etc.	54/54	32/76	51.3 ± 8.6	zoledronic acid+CKI	zoledronic acid	28	①②	Not described
Bai Zhaoqin 2016	Breast cancer	32/31	63/0	50.5 ± 8.5/51.1 ± 8.2	zoledronic acid+CKI	zoledronic acid	20	①	Random number table
Zhang Chao 2015	Lung cancer, breast cancer, etc.	37/36	30/43	51.8 ± 5.7/51.2 ± 6.1	zoledronic acid+CKI	zoledronic acid	14	①②	Not described
Li Yajun 2013	Nasopharyngeal Carcinoma	30/30	13/47	54.2 ± 4.6/55.1 ± 5.2	zoledronic acid+CKI	zoledronic acid	28*3	①②	Random sampling
Wu Qingzhen 2017	Lung cancer, breast cancer, stomach cancer, etc.	45/45	48/42	58.37 ± 3.44/58.94 ± 3.56	zoledronic acid+CKI	zoledronic acid	28	①	Not described

① The incidence of total effective rate. ② Adverse effects for pain relief.

#### 3.1.2 Meta-analysis of overall pain relief rate

**Figure 5:**
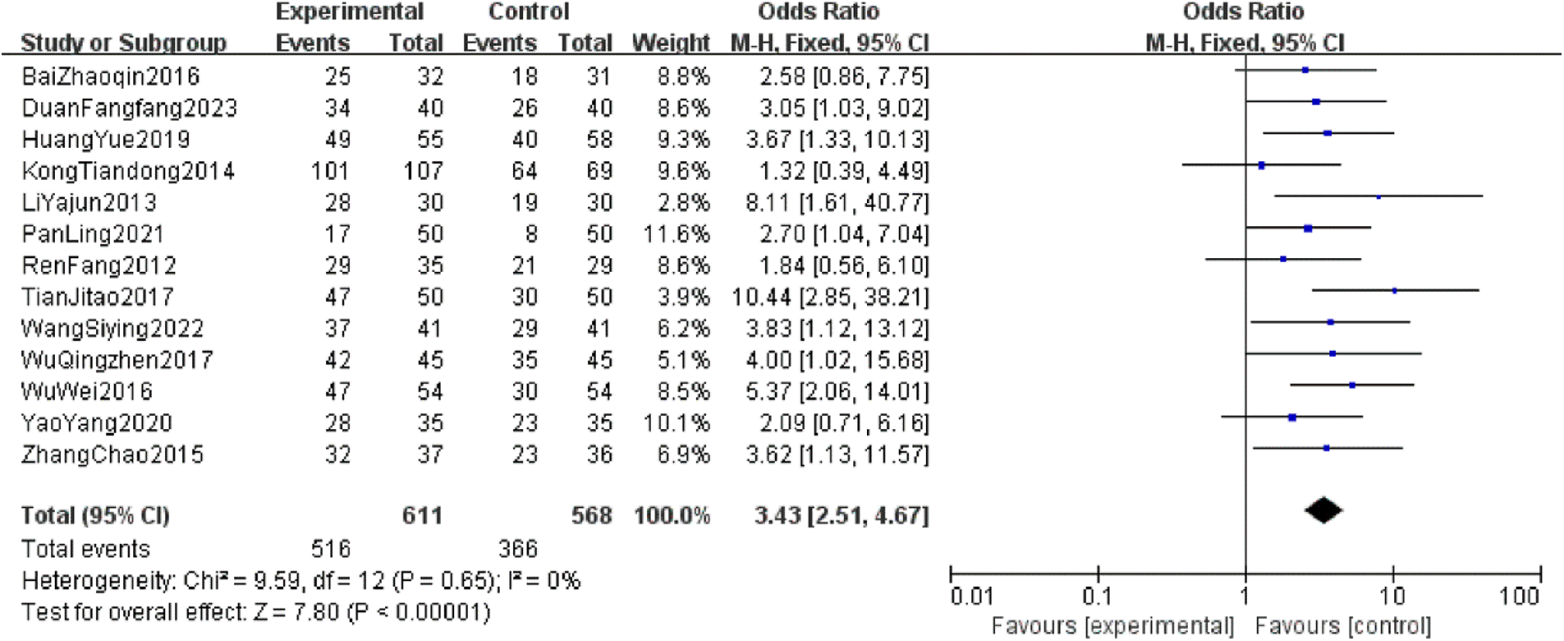
Forest plot of total pain relief rate in two groups.

A total of 13 RCTs comprising 1,179 patients were included. The heterogeneity test showed *P* > 0.05 and *I*^2^ = 0%, indicating no significant heterogeneity; thus, a fixed-effect model was used for the analysis. The meta-analysis demonstrated that the overall effect of pain relief for CKI combined with zoledronic acid had an OR of 3.43 (95% CI: 2.51–4.67, *P* < 0.0001), with a statistically significant difference. This suggests that the combination of CKI with zoledronic acid is more effective in treating bone metastatic cancer pain compared to zoledronic acid alone (Figure 5).

#### 3.1.3 Meta-analysis of adverse reaction incidence

**Figure 6:**
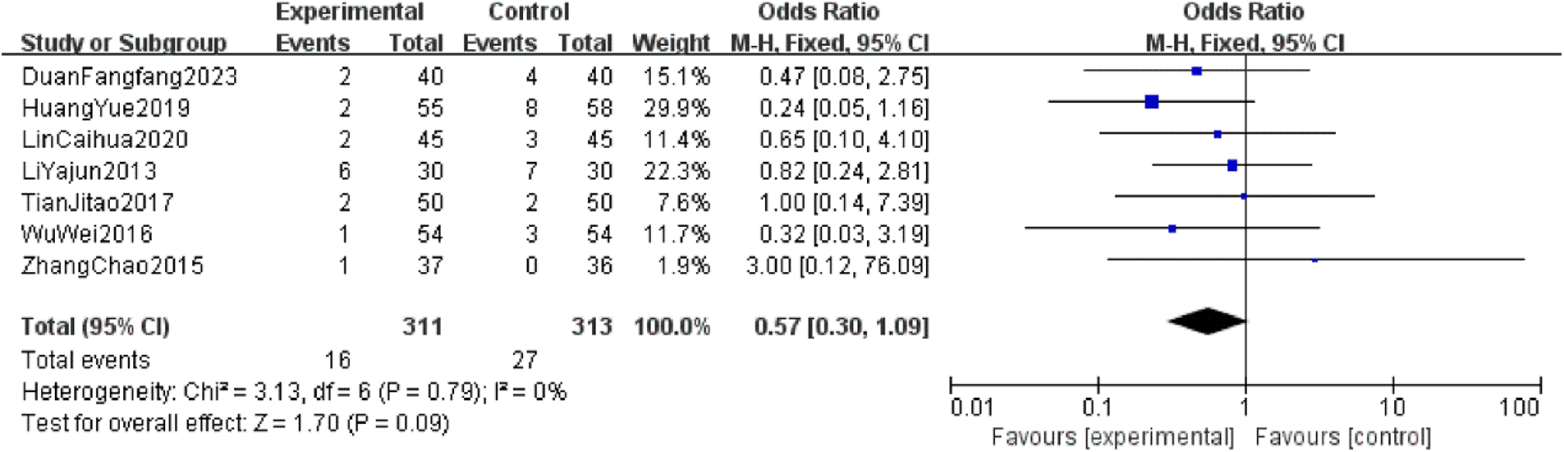
Forest plot of fever incidence rates in two groups.

**Figure 7:**
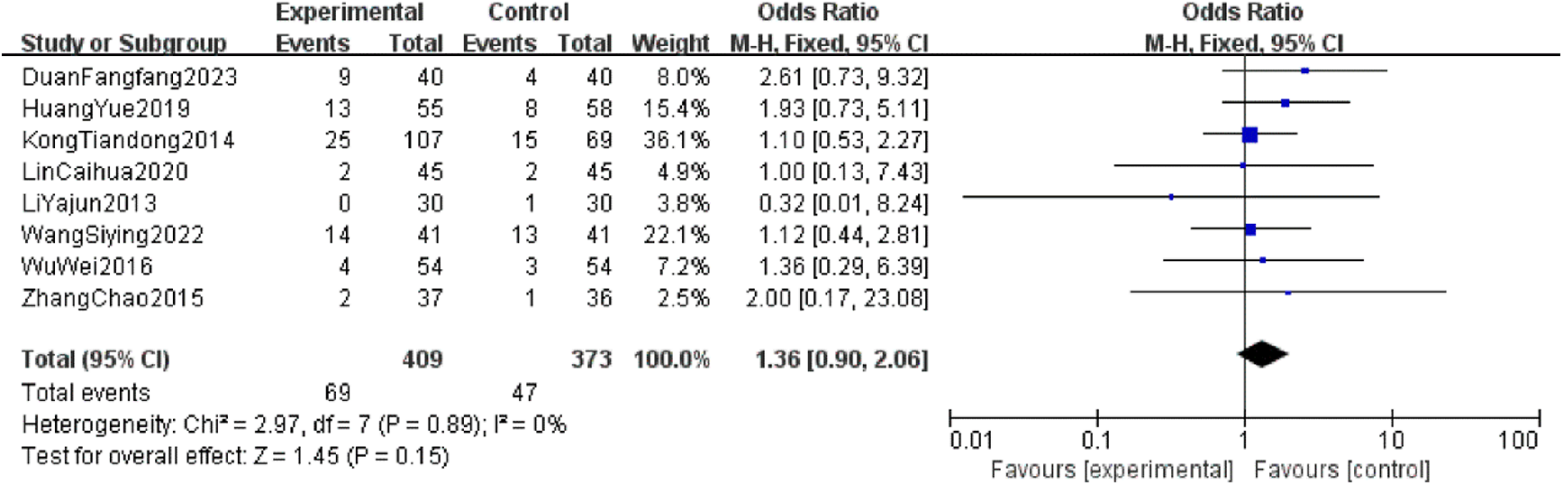
Forest plot of incidence of gastrointestinal adverse reactions in two groups.

**Figure 8:**
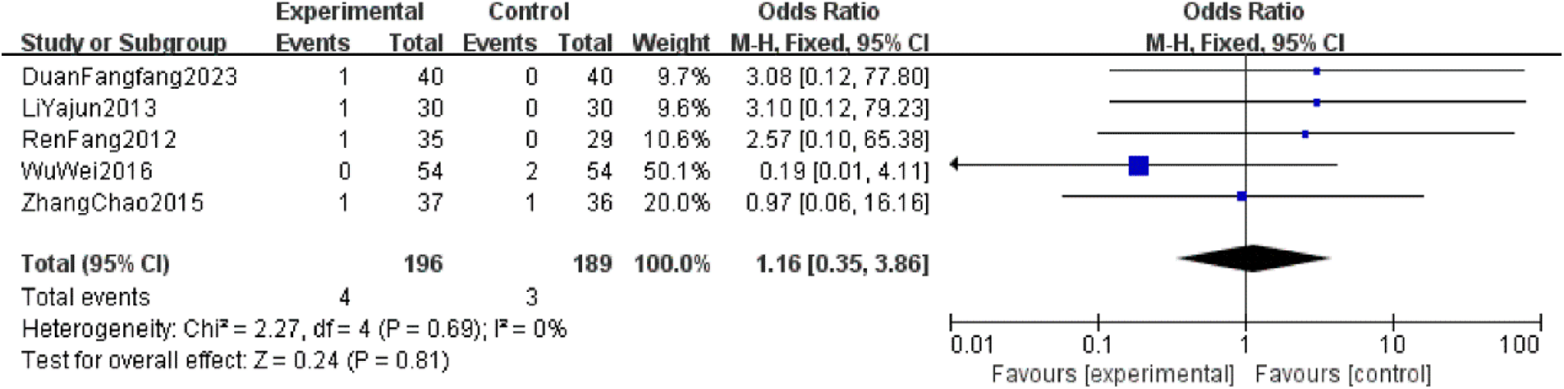
Forest plot of incidence of adverse skin reactions in two groups.

The heterogeneity tests for fever, gastrointestinal adverse reactions, and skin adverse reactions all resulted in *P* > 0.05 and *I*^2^ = 0%, indicating no significant heterogeneity. Thus, a fixed-effect model was used for analysis. The combined OR values crossed the line of no effect, and the *P*-values exceeded 0.05, suggesting no statistically significant difference in the incidence of major adverse reactions between the two intervention groups. This indicates that CKI has a favorable safety profile (Figures 6–8).

#### 3.1.4 Publication bias

**Figure 9:**
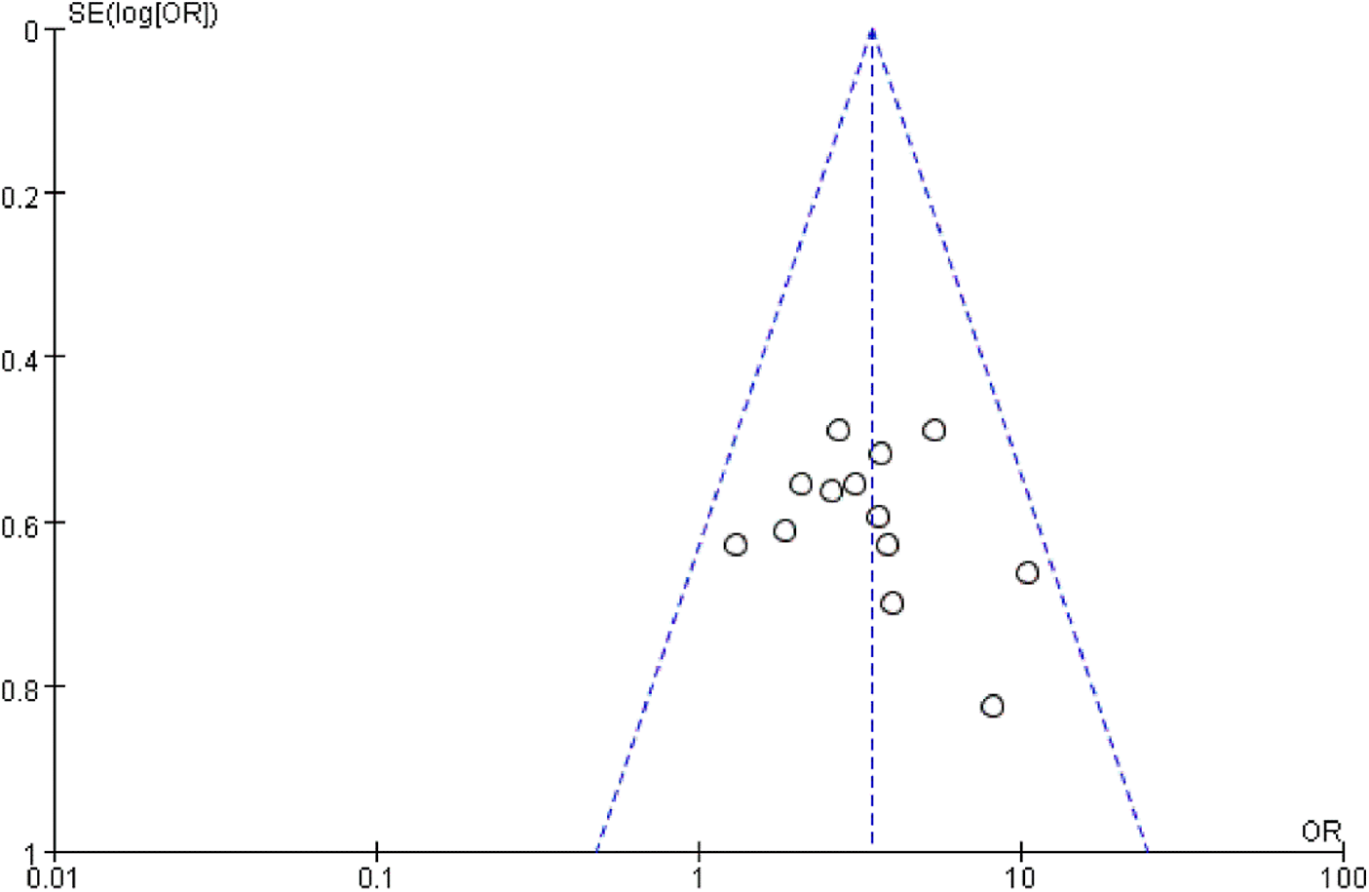
Funnel plot of comparison.

A funnel plot was generated to analyze publication bias for the overall pain relief rate in the combination treatment of CKI and zoledronic acid for bone metastatic cancer pain. The results showed a roughly symmetrical distribution, indicating no significant publication bias in the current studies (Figure 9).

### 3.2 Economic analysis results

#### 3.2.1 Cost-effectiveness analysis results

Based on the weights of each study from the forest plot (Figure 5), the pain relief rates were weighted to calculate the overall effectiveness rate. The results indicate that the overall effectiveness rate for the treatment group is 0.81, while for the control group it is 0.62. Thus, the effect values for the two groups are 0.81 and 0.62, respectively. The costs and relevant parameters required for the model are presented in Table 2.

**Table 2:** Costs and related parameters.

Parameters	Mean value	Lower limit	Upper limit	Distribution
Unit price of Compound Kushen injection (5 ml) (Yuan)	32	25.4	38.6	Gamma
Unit price of zoledronic acid (Yuan)	253.6	202.9	304.3	Gamma
Duration of Compound Kushen usage	28	22	36	Uniform
Duration of zoledronic acid usage	2	1	3	Uniform
Daily usage of Compound Kushen injection	4	2	6	Uniform
Utility values of the test group	0.81	0.65	0.97	Beta
Utility values of the control group	0.62	0.50	0.74	Beta

The cost-effectiveness analysis results are shown in Table 3. The incremental effect is 0.19, and the incremental cost is ¥3,584, resulting in an ICER of ¥18,863.16, which is below the WTP threshold of ¥39,218. Therefore, using the per capita disposable income of 2023 as the WTP threshold, the combination of CKI and zoledronic acid is more economically viable compared to using zoledronic acid alone.

**Table 3:** Basic analysis results.

Therapeutic regimen	Effect	Incremental effect	Cost (Yuan)	Incremental cost	ICER
Compound Kushen injection + zoledronic acid	0.81	0.19	4,091.2	3,584	18,863.16
zoledronic acid	0.62		507.2		

#### 3.2.2 Sensitivity analysis

**Figure 10:**
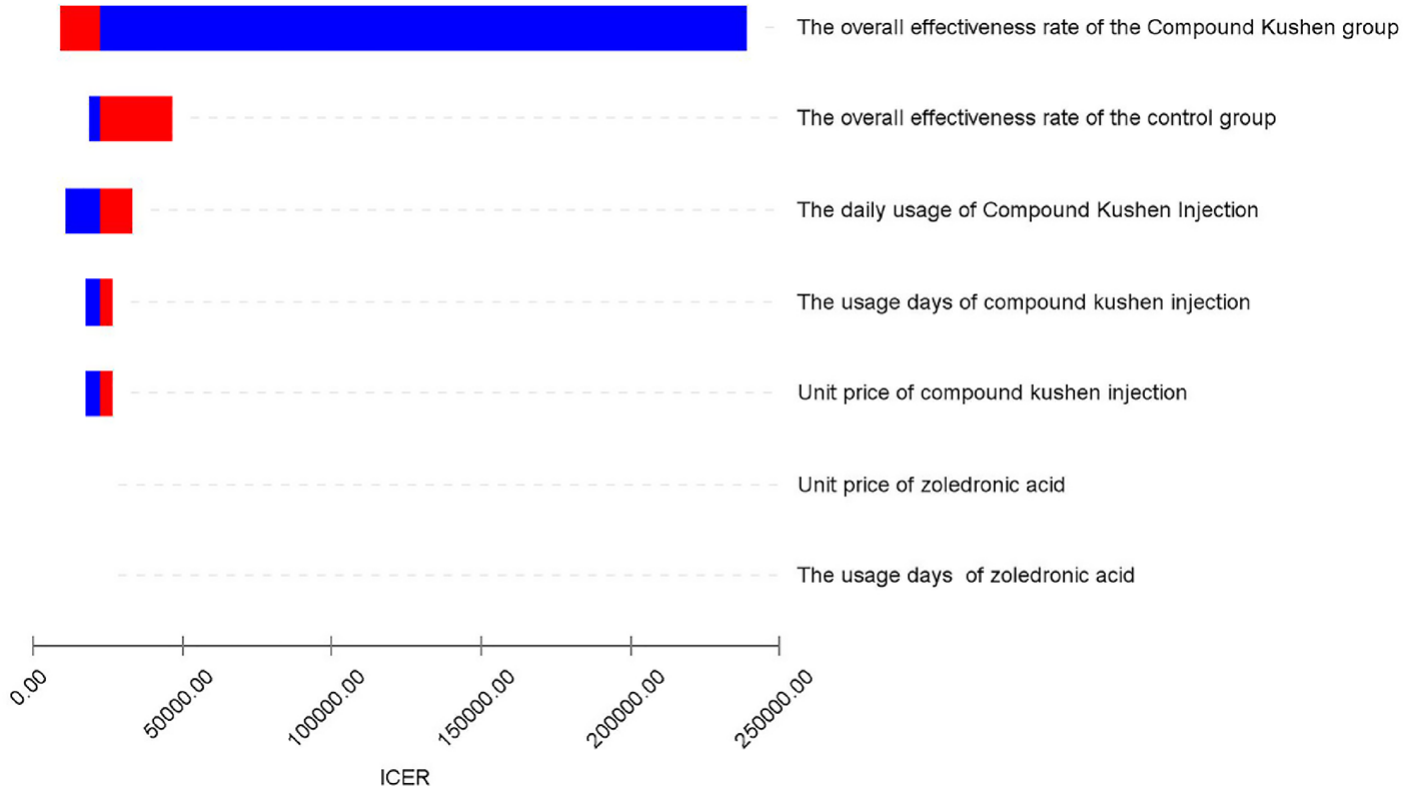
Tornado chart of one-way sensitivity analysis.

The tornado diagram (Figure 10) illustrates the results of the one-way sensitivity analysis. The three parameters with the greatest impact on the results are the overall effectiveness rate of the CKI group, the overall effectiveness rate of the control group, and the daily usage of CKI. Other factors, such as the duration of CKI usage, the unit price of CKI, the unit price of zoledronic acid, and the duration of zoledronic acid usage, have relatively minor effects on the results.

**Figure 11:**
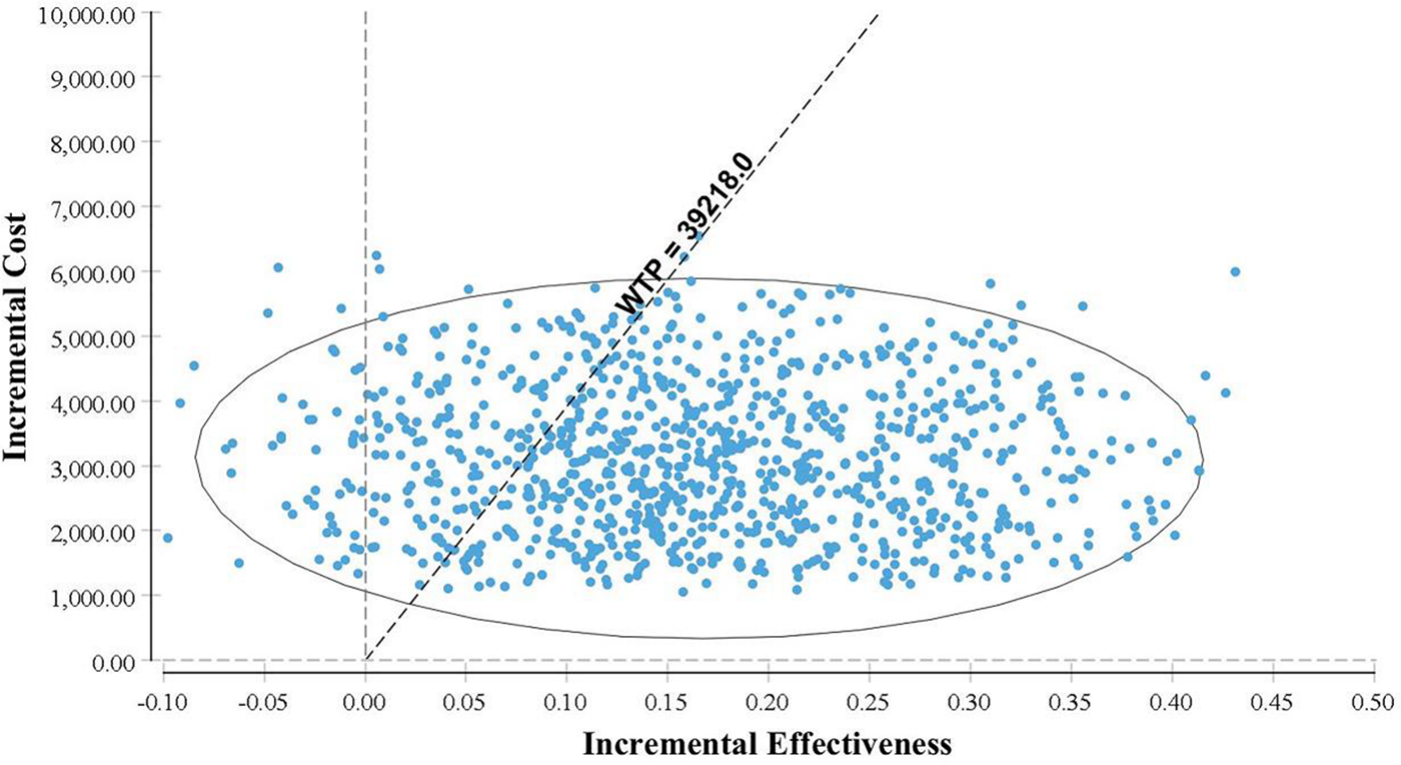
Incremental CE scatter plot.

**Figure 12:**
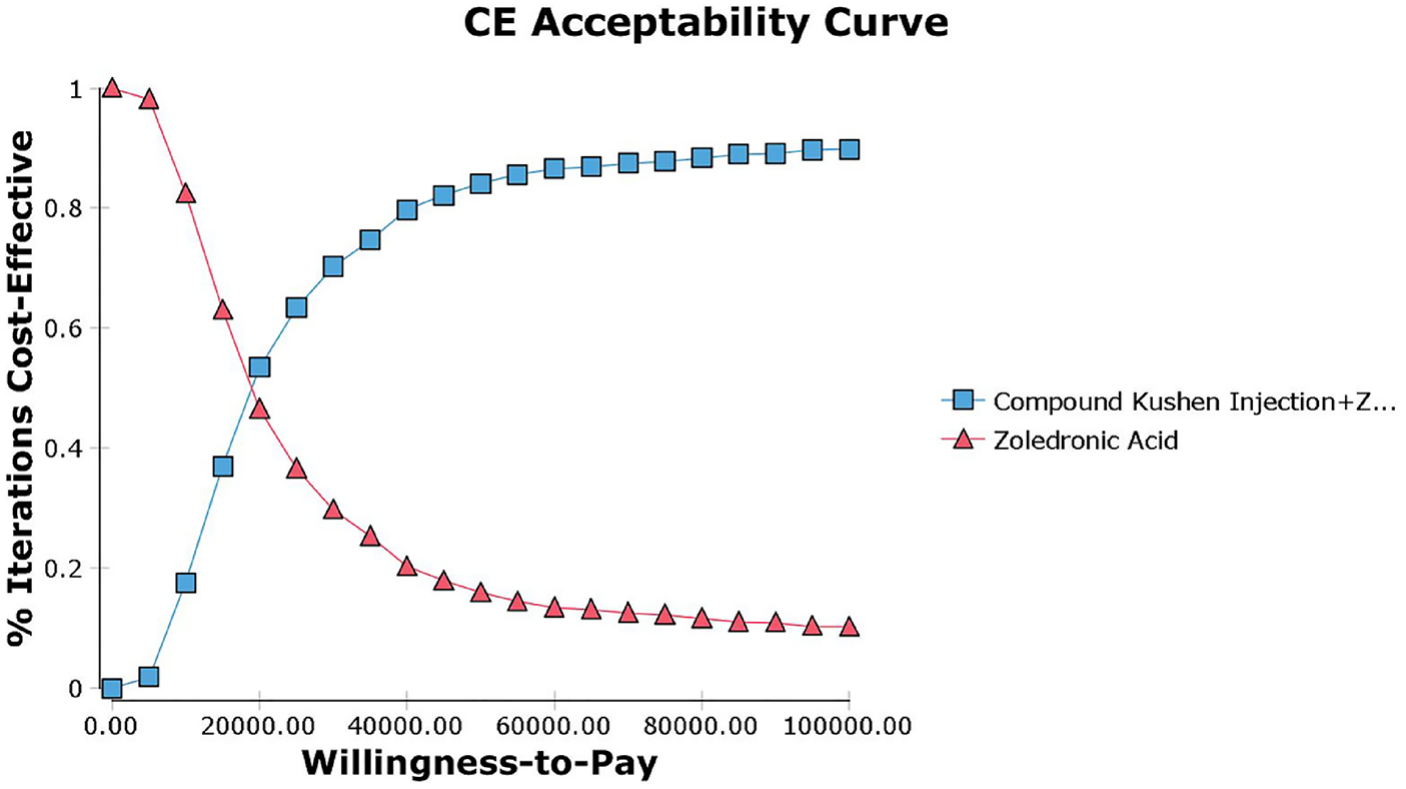
CE acceptability curve.

The probabilistic sensitivity analysis conducted through 1,000 rounds of Monte Carlo simulation indicates that a significant number of points in the incremental cost-effectiveness scatter plot cluster below the WTP threshold of ¥39,218 (Figure 11). This suggests that, at the payment threshold of ¥39,218, the combination of CKI and zoledronic acid provides greater health benefits compared to using zoledronic acid alone. The cost-effectiveness acceptability curve indicates that when the WTP threshold exceeds ¥18,863.16, the probability of the economic viability of the combination therapy gradually increases, further supporting the economic advantage of combining CKI with zoledronic acid (Figure 12).

## 3 Discussion

This study focuses on the total effective rate of pain relief as the primary outcome indicator for efficacy analysis. The meta-analysis results confirmed that the combination of zoledronic acid and CKI is more effective in treating bone cancer pain than using zoledronic acid alone (OR = 3.43, 95% CI: 2.51–4.67, *P* < 0.0001). Currently, bisphosphonates are the main drugs for treating bone cancer pain, with zoledronic acid being the representative. It works primarily by inhibiting osteoclast maturation and inducing their apoptosis, thus preventing their adhesion and aggregation in bone resorption areas while also repairing osteolytic bone damage, effectively preventing and delaying bone-related pain (26). However, clinical practice has shown that the efficacy of bisphosphonate monotherapy for bone cancer pain is often insufficient. According to the principles of comprehensive cancer treatment, combining multiple therapeutic approaches, such as integrating TCM, can significantly improve treatment outcomes and ensure patients' quality of life (27).

Unlike Western medicine's single-target mechanisms, TCM emphasizes holistic regulation and multi-pathway interactions, providing TCM with unique advantages in cancer treatment. CKI is a traditional Chinese medicinal preparation widely used in oncology, containing key active ingredients such as matrine, oxymatrine, and poria. Although the exact analgesic mechanisms of CKI remain unclear, it is believed to reduce the central nervous system's sensitivity to pain stimuli and activate *κ*-opioid receptors in the spinal cord, thereby exerting analgesic effects (28, 29). Studies have demonstrated that the combination of CKI and zoledronic acid has a synergistic effect in alleviating pain caused by bone metastases, significantly enhancing pain relief, improving patients' quality of life, and increasing their tolerance to chemotherapy and radiotherapy.

A safety evaluation was conducted by comparing the incidence of adverse reactions between patients treated with the combination of CKI and zoledronic acid and those treated with zoledronic acid alone. The meta-analysis showed no statistically significant differences between the two groups in terms of fever (OR = 0.57, 95% CI: 0.30–1.09, *P* = 0.09), gastrointestinal adverse reactions (OR = 1.36, 95% CI: 0.90–2.06, *P* = 0.15), and skin reactions (OR = 1.16, 95% CI: 0.35–3.86, *P* = 0.81), confirming that CKI has a favorable safety profile with no obvious adverse reactions. A literature-based analysis of the adverse reactions (ADRs) and safety of CKI (30) included 13 studies involving a total of 76 patients. The occurrence of ADRs was predominantly observed in individuals over 40 years old, with a higher incidence in females (41 cases) compared to males (35 cases). The occurrence of ADRs may be associated with factors such as daily dosage, solvent, concomitant medications, and patient history of allergies. The highest risk period for ADRs is within the first 30 min of the initial infusion of CKI. Clinically, ADR manifestations mainly involved gastrointestinal reactions and systemic damage. All 76 cases of ADRs were resolved or significantly improved after discontinuation of the drug and symptomatic treatment, with no fatal cases reported. Therefore, in clinical practice, it is crucial to inquire about patients' allergy histories, strictly follow the recommended solvents and dosages in the drug's instructions, avoid inappropriate drug combinations, and closely monitor patients during treatment to minimize the risk of adverse reactions while treating the disease.

Our findings are consistent with previous studies. For instance, Guo et al. (31) found in a systematic review and meta-analysis that CKI as an adjunctive therapy helps alleviate cancer-related pain and improves patients’ quality of life. Additionally, Yang et al. (32) discovered that CKI modulates tumor-associated macrophage-mediated immunosuppression, enhancing the sensitivity of hepatocellular carcinoma to sorafenib. These studies support the synergistic role of CKI in cancer treatment, aligning with our meta-analysis and economic evaluation results, thereby further demonstrating the effectiveness and economic feasibility of combining CKI with zoledronic acid in managing bone metastasis-induced cancer pain.

Internationally, treatments for bone metastasis-induced pain also include medications such as Denosumab. Existing studies have evaluated the cost-effectiveness of Denosumab compared to bisphosphonate drugs like zoledronic acid within developed countries' healthcare systems. The results indicate that while Denosumab can reduce the incidence of bone-related events to some extent, its cost is significantly higher than that of bisphosphonates (33). In contrast, our study found that the incremental cost-effectiveness ratio (ICER) of combining CKI with zoledronic acid was significantly below the Chinese per capita disposable income threshold (¥39,218), demonstrating that this combination therapy is economically viable within the context of China's healthcare system. Future research could incorporate economic data for Denosumab or other non-TCM treatment options in China to facilitate indirect comparative analyses, thereby providing a comprehensive evaluation of the cost-effectiveness of different treatment strategies. This study conducted a cost-effectiveness evaluation of the combination of CKI and zoledronic acid in treating bone cancer pain induced by malignant tumors from the perspective of the Chinese healthcare system. Assuming a willingness-to-pay (WTP) threshold of ¥39,218, the per capita disposable income in China in 2023, the combination of CKI and zoledronic acid proved to be more cost-effective, with an ICER of ¥18,863.16. Sensitivity analyses indicated that the results were relatively stable.

Due to the lack of large-scale RCTs or relevant real-world studies on CKI, key parameters required for the economic model were unavailable. Therefore, this study utilized clinical efficacy and adverse reaction rates from a meta-analysis for cost-effectiveness analysis (34). However, the studies included in the meta-analysis generally suffered from low quality, primarily due to the absence of randomization and blinding, which may introduce bias in the results. Consequently, there is an urgent need for high-quality studies, including multicenter, large-sample RCTs and real-world studies, to more accurately assess the cost-effectiveness of this combination therapy and provide more comprehensive and reliable evidence.

Nonetheless, this study has several limitations. Primarily, the clinical studies included in the meta-analysis were all Chinese literature, consisting of small-sample, single-center studies with relatively low quality, which may affect the reliability of the results. Additionally, due to the lack of relevant clinical data on the long-term use of CKI, this study employed a decision tree model to evaluate the cost-effectiveness of short-term treatment. Short-term outcomes do not adequately reflect the long-term disease progression, efficacy, and cost-effectiveness for cancer patients. Furthermore, during our literature search, we included major international databases such as PubMed, EMBASE, and the Cochrane Library, but did not identify any high-quality English RCTs that met the inclusion criteria. This may be attributed to the limited international application of CKI or the absence of relevant studies published in international journals. Consequently, this limitation restricts the international applicability of our study findings. Future research should aim to promote the clinical application of CKI internationally and incorporate high-quality international studies to enrich the existing evidence base.

In summary, the combination of CKI and zoledronic acid for the treatment of malignant tumor-induced bone metastatic pain shows promise for further clinical research and dissemination. Additionally, it is anticipated that the economic evaluation can be enhanced based on real-world data.

## Data Availability

The original contributions presented in the study are included in the article/Supplementary Material, further inquiries can be directed to the corresponding author.
